# Flow Cytometry Assessment of In Vitro Generated CD138**^+^** Human Plasma Cells

**DOI:** 10.1155/2014/536482

**Published:** 2014-02-09

**Authors:** Rayelle Itoua Maïga, Jennifer Lemieux, Annie Roy, Carl Simard, Sonia Néron

**Affiliations:** ^1^Héma-Québec, Ingénierie cellulaire, Recherche et Développement, 1070 Avenue des Sciences-de-la-Vie, Québec, QC, Canada G1V 5C3; ^2^Université Laval, Faculté des Sciences et de Génie, Département de Biochimie et Microbiologie, 1045 Avenue de la Médecine, Québec, QC, Canada G1V 0A6

## Abstract

The in vitro CD40-CD154 interaction promotes human B lymphocytes differentiation into plasma cells. Currently, CD138 is the hallmark marker enabling the detection of human plasma cells, both in vitro and in vivo; its presence can be monitored by flow cytometry using a specific antibody. We have developed a culture system allowing for the differentiation of memory B lymphocytes. In order to detect the newly formed plasma cells, we have compared their staining using five anti-CD138 monoclonal antibodies (mAbs). As a reference, we also tested human cell lines, peripheral blood mononuclear cells, and bone marrow samples. The five anti-CD138 mAbs stained RPMI-8226 cells (>98%) with variable stain index (SI). The highest SI was obtained with B-A38 mAb while the lowest SI was obtained with DL-101 and 1D4 mAbs. However, the anti-CD138 mAbs were not showing equivalent CD138^+^ cells frequencies within the generated plasma cells. B-A38, B-B4, and MI-15 were similar (15–25%) while DL-101 mAb stained a higher proportion of CD138-positive cells (38–42%). DL-101 and B-A38 mAbs stained similar populations in bone marrow samples but differed in their capacity to bind to CD138^high^ and CD138^lo^ cell lines. In conclusion, such cellular fluctuations suggest heterogeneity in human plasma cell populations and/or in CD138 molecules.

## 1. Introduction

Syndecan-1 molecules, also named CD138, are a type I transmembrane proteoglycans, which may act as coreceptors in cell adhesion and regulate several cellular functions [1]. In human lymphomas, CD138 molecules are present in isoforms of distinct molecular weight, which is dependent upon the glycosaminoglycan chains covalently attached to the core protein [2]. The CD138 marker is used in flow cytometry to identify normal or malignant plasma cells (reviewed in [3]). Several monoclonal antibodies are commercially available to detect CD138^+^ cells; the most currently used are the clones B-A38, B-B4, MI-15, ID4, and DL-101. These monoclonal antibodies are all of mouse origin but they differ in the source of CD138 antigen for immunization. In fact, U266, XG-1, or TH-1 cells, all CD138^+^ cell lines, were used as immunogens for B-A38, B-B4 [4], MI-15 [5], and 1D4, while recombinant syndecan-1 was used for DL-101 [6]. The B-B4 epitope is located within amino acids 90 and 93 of the core protein [7], which is far from the heparin sulfate and chondroitin sulfate attachments sites. The binding of B-B4 on U266 cells can be inhibited by MI-15 and 1D4 in competitive assays [2]. However, the B-B4 epitope appears distinct from the epitopes of MI-15 [5] and 1D4 when tested on lymphoma samples [8]. Finally, DL-101 monoclonal antibody reacts with the native ectodomain of syndecan-1 [6].

CD40 activation of human B lymphocytes can lead to the in vitro generation of CD138^+^ plasma cells [9–13]. Such in vitro models are essential for the better understanding of B lymphocyte terminal differentiation. Since CD138 staining is crucial for the detection of newly formed CD138^+^ plasma cells, we have investigated the binding efficiency of five anti-CD138 monoclonal antibodies in flow cytometry analysis by comparing their capacity to detect human plasma cells.

## 2. Material and Methods

### 2.1. Human Cell Lines and Bone Marrow Samples

RPMI-8226 cell line was purchased from DSMZ (Deutsche Sammlung von Mikroorganismen und Zellkulturen GmbH, Braunschweig, Germany) while Ramos and SKW 6.4 cell lines were obtained from American Type Culture Collection (ATCC, Rockville, MD) and cultured in IMDM or RPMI supplemented with 10% fetal bovine serum (FBS) (Hyclone, Logan, UT, USA). All cell lines were mycoplasma-free.

Bone marrow samples from healthy human were obtained from (Lonza, Walkersville, USA). These samples were received frozen and kept in liquid nitrogen vapor until flow cytometry analysis.

### 2.2. Human Peripheral Blood Mononuclear Cells and Switched-Memory B Lymphocytes

This study has been approved by Héma-Québec's Research Ethics Committee and every regular platelet donor who agreed to participate in this study has signed an informed consent after the nature and possible consequences of the studies had been fully explained. Leukoreduction system (LRS) chambers from Trima Accel collection systems (Gambro BCT, Lakewood, CO, USA) were used to isolate peripheral blood mononuclear cells (PBMCs) as described previously [14]. CD19^+^ B lymphocytes were isolated from PBMNCs by negative selection using EasySep CD19 cocktail and EasySep custom cocktail containing antibodies directed against IgD and IgM following manufacturer's instructions (Stem Cell Technologies, Vancouver, BC, Canada) [15]. B lymphocytes' purity, as determined by flow cytometry, was higher than 95%.

### 2.3. Human B Lymphocytes Culture

B lymphocytes were seeded at 2 to 3 × 10^5^ cells/mL in 6-well Primaria plates (BD Biosciences, Mississauga, Canada) in the presence of 4 to 6 × 10^4^ cells/mL *γ*-irradiated CD154^+^ L4.5 cells [11]. The cells were cultured in IMDM supplemented with 10% ultra low IgG FBS containing 10 *μ*g/mL insulin, 5.5 *μ*g/mL transferrin, 6.7 ng/mL sodium selenite (all from Invitrogen, Burlington, ON, Canada), and a mix of cytokines, namely, 5 ng/mL IL-2 (~50 U/mL), 40 ng/mL IL-10 (~20 U/mL) (both from PeproTech, Rocky Hill, NJ, USA), and 3.5 ng/mL IL-4 (100 U/mL; R&D Systems, Minneapolis, MN, USA) [15]. On day 19, IL-6 (12.5 ng/mL) (PeproTech) was added to the medium and the cells were transferred in the presence of 1 to 1.5 × 10^4^ cells/mL *γ*-irradiated CD154^+^ L4.5 cells. This second step was used to induce their differentiation into plasma cells [11] for a supplemental 5 culture days. Cell counts and viability were evaluated in triplicate by Trypan Blue exclusion using a hemocytometer.

#### 2.3.1. Flow Cytometry Analysis

FITC conjugated anti-CD3 (UCHT1), PE-Cy7 conjugated anti-CD38 (HIT2), APC conjugated anti-CD14 (M5E2), and PE-Cy7 conjugated anti-CD19 (SJ25C1) were all from Becton Dickinson Canada (Mississauga, ON, Canada). Cells were also stained with APC-efluor 780 conjugated anti-CD19 (HIB19, eBioscience, San Diego, CA, USA), Krome Orange anti-CD45 (J33, Beckman Coulter, Mississauga, ON, Canada), and Alexa 647 conjugated CD138 (B-A38, AbD Serotec, Raleigh, NC, USA). Five anti-CD138 monoclonal antibodies were used, namely, PE-conjugated B-A38 (Abcam, Cambridge, MA, USA), B-B4 (AbD Serotec), DL-101 (BioLegend, San Diego, CA, USA or eBioscience), and MI-15 (Becton Dickinson Canada, Mississauga, ON, Canada), as well as FITC-conjugated 1D4 (eBioscience). Dead cells were excluded by Pacific Blue or Sytox Blue staining (Molecular Probes, Life technologies, Burlington, ON, Canada). All antibodies were used following manufacturer's instructions; more specifically for each 100 *μ*L containing about 1 × 10^6^ cells, we were using 20 *μ*L of PE-conjugated 1D4, B-A38, B-B4, and MI-15 and 5 *μ*L of Alexa 647 conjugated B-A38. The volume for PE-conjugated DL-101 from eBioscience was 5 *μ*L and that for BioLegend was 20 *μ*L. Titration of DL-101 and B-A38 PE conjugated antibodies was done using RPMI-8226 cells. Staining was done for 15 to 30 minutes at room temperature in PBS and, when indicated, cells were fixed with 2% formaldehyde and analyzed immediately or kept overnight at 4°C. For each sample, ≥10,000 events were gated on the FSC/SSC to analyse the cells using a Partec CyFlow ML flow cytometer (Swedesboro, NJ, USA) or Accuri C6 flow cytometer and data were subsequently analyzed using FCS Express 4.0 software (De Novo software, Los Angeles, CA, USA). Stain index was calculated using the following formula: median fluorescence intensity (MFI) obtained from the given mAb – MFI from unstained cells divided by 2 times the SD of the MFI from unstained cells. Bone marrow samples were thawed in PBS containing 50% fetal bovine serum. Sytox Blue was used to gate the viable cells in combination with CD45, CD19, and CD138 mAbs. Bone marrow cells were not fixed and analyses were done on the same day.

### 2.4. Statistical Analysis

When indicated, the mean values ± standard deviations or standard error of the mean (SEM) were calculated. Gaussian distribution and variance were used to choose the adequate statistical test used to compare data as specified for each analysis. See each figure legends for details on the specific tests used, which were done using GraphPad InStat software (GraphPad software Inc. San Diego, USA).

## 3. Results

### 3.1. Comparison of Anti-CD138 Antibodies on RPMI-8226 Cells

RPMI-8226 is a human myeloma cell line, which expresses high levels of CD138 molecules [16]. This CD138^+^ cell line was stained with the five mAbs and immediately analyzed by flow cytometry with or without fixation or after an overnight period following fixation (Table 1). All anti-CD138 monoclonal antibodies staining resulted, as expected, in 100% CD138^+^ RPMI-8226 cells. However, the stain index comparisons showed that B-A38 had the highest stain index value; B-B4 and MI-15 were intermediate whereas DL-101 and 1D4 were showing the less efficient staining. Preliminary tests, using the reagent listed in Table 1, showed that in contrast to 1D4 mAb DL-101 mAb was however able to stain a large proportion of cultured human B lymphocytes (data not shown). Furthermore, the SI values on fixed and unfixed cells showed that the staining with the PE-conjugated antibody DL-101 was the most stable.

### 3.2. Variations in Detection of In Vitro Generated Plasma Cells

**Figure 1:**
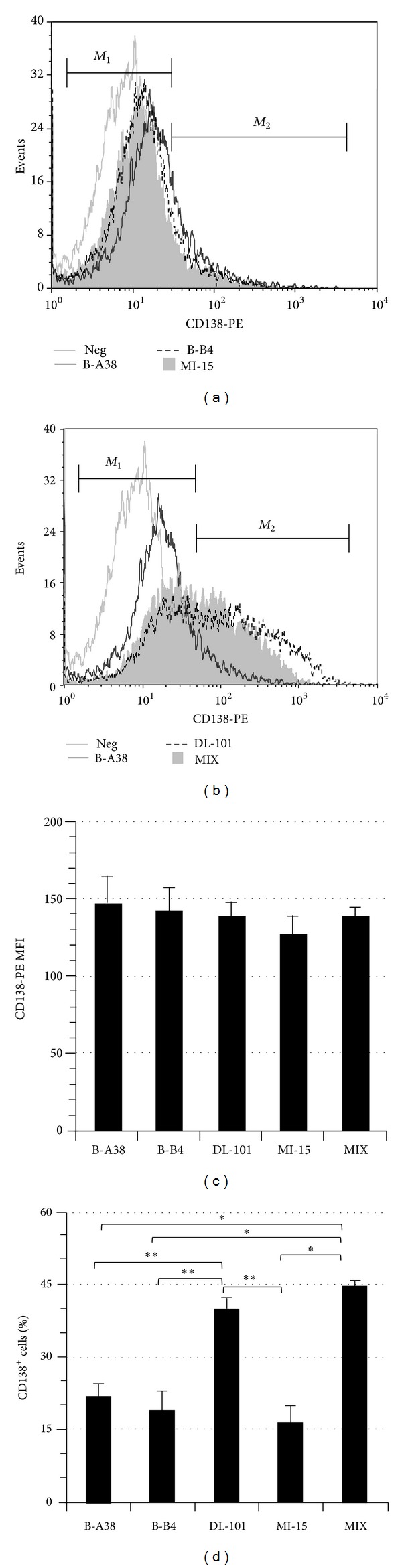
CD138^+^ cells staining in newly generated plasma cells. Switched memory B lymphocytes were cultured for 24 days as described in M&M. The cells were stained with one of four PE-conjugated anti-CD138 mAbs, namely, B-A38, B-B4, DL-101, and MI-15, or with a mix of B-A38 and DL-101 (MIX). The cells were fixed, kept in the dark, and analyzed 18 h to 20 h later. CD138 expression profile obtained with B-A38 for one sample out of six independent experiments is compared in (a) with B-B4 and MI-15 staining and in (b) with DL-101 and the mix of DL-101 and B-A38 (MIX). (c) The median fluorescence intensity (MFI) for each CD138 staining condition is presented as the mean ± S.E.M. for B-B4 and MI-15 staining (*n* = 6) and for B-A38, DL-101, and the MIX staining (*n* = 10) as observed for in vitro generated plasma cells. No significant differences were obtained for MFI values for any possible comparisons. (d) The CD138^+^ cells frequency in the viable cells (Pacific blue negative) is presented as the mean ± S.E.M for each staining condition (*n* = 6 or *n* = 10 as in panel (c)). Multiple comparisons were performed using a Bonferroni correction. Differences were observed between DL-101 and BA-38, B-B4, and MI-15 (indicated by **; *P* < 0.001) as well as the MIX and BA-38, B-B4, and MI-15 (indicated by *; *P* < 0.001).

Human switched-memory B lymphocytes can be expanded and differentiated into antibody secreting cells [15], which express CD138 [17]. We used a two-step culture model starting with a 19-day expansion, followed by 5 days in conditions favorable to differentiation. On day 24, the differentiated cells were used to test the staining capacity of B-A38, B-B4, DL-101, and MI-15 PE-conjugated anti-CD138 antibodies on de novo generated plasma cells (Figure 1). We also tested the combination of B-A38 and DL-101 (MIX). These tests were performed using the “worst case scenario protocol” with fixation and analysis 18 h to 20 h apart. Representative profiles of CD138^+^ cells for all staining conditions are presented in (a) and (b); the profile for B-A38 staining is included in panels (a) and (b) to facilitate comparison. No differences were observed for CD138 mean fluorescence intensity (MFI) inside the CD138^+^ cell populations indicating a similar binding strength for their specific target (Figure 1(c)). The use of B-A38, B-B4, and MI-15 showed a frequency of CD138^+^ cells varying from 23% ± 9% to 16% ± 8% (Figure 1(d)), which was as previously observed in similar culture conditions with CD19^+^ B cells [11, 17]. However, when DL-101 was used alone or in combination with B-A38 (MIX) (Figures 1(b) and 1(d)), the proportion of CD138^+^ cells was always twofold higher than staining with B-A38, B-B4, or MI-15 alone (*P* < 0.001). Such increased proportion of CD138^+^ cells related to DL-101 staining was also observed on day 19 within cultured B lymphocytes (data not shown) suggesting that DL101 could detect a supplemental population of emerging plasma cells.

### 3.3. DL-101 Binding in Human Plasma Cells

**Figure 2:**
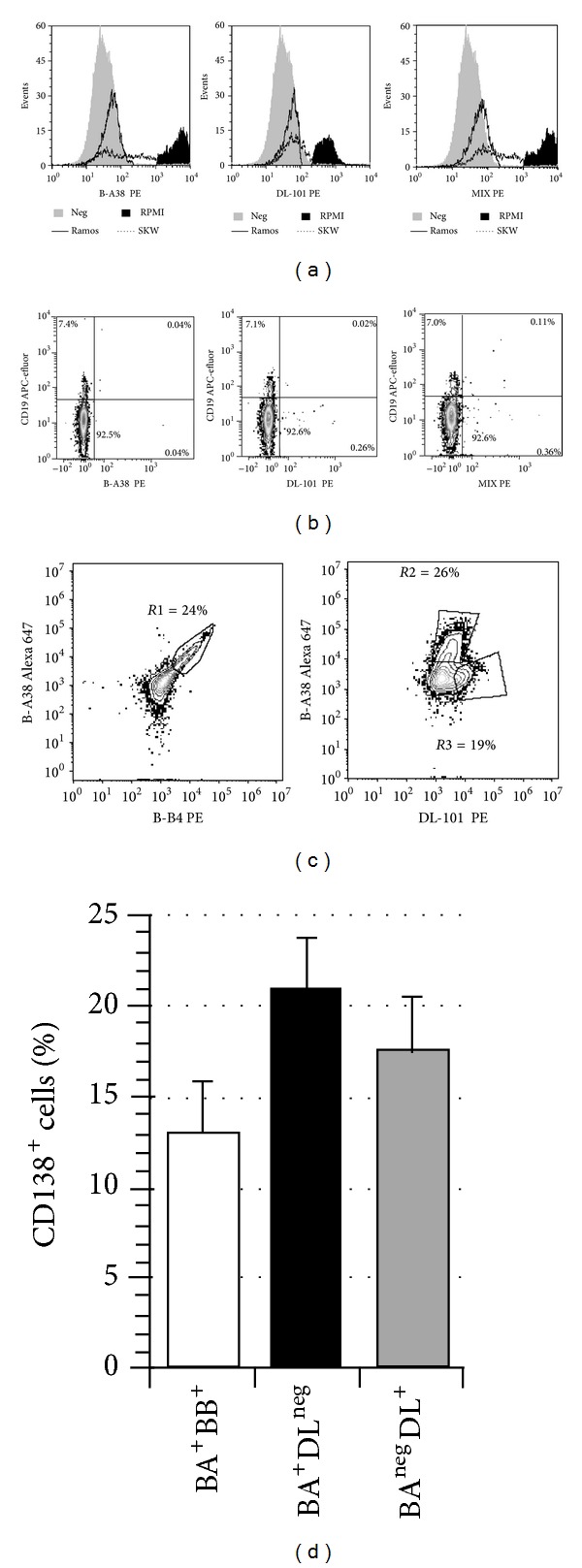
DL-101 binding on human cells lines, blood CD19^+^ cells, and in vitro plasma cells. RPMI-8226, SKW6.4, and Ramos cell lines were mixed in equal proportion and stained with PE-conjugated B-A38 and DL-101 alone or combined (MIX). The staining profiles are presented for the three conditions (a). The same three anti-CD138 conditions were also used on four independent samples of blood mononuclear cells. (b) Sytox Blue, CD45, and CD19 were first used to gate the viable CD19^+^CD45^+^ cells which were then analyzed for CD138 expression. (c) Differentiated B lymphocytes, prepared as in Figure 1, were simultaneously stained with PerCP-conjugated CD19 antibodies, APC-efluor-conjugated B-A38, and either PE-conjugated B-B4 or DL-101. Analyses were done on gated CD19^+^ viable cells. An example representative of 5 independent experiments is shown. R1 gate included double-positive cells for B-A38 (BA) and B-B4 (BB) mAbs while R2 and R3 gates included single-positive cells for B-A38 (BA) or DL-101 (DL) mAbs, respectively. (d) The gates established in (c), namely, BA^+^BB^+^ (R1), BA^+^DL^neg^ (R2), and BA^neg^DL^+^ (R3), were used to establish the proportion of these CD138^+^ cells subsets for five independent B lymphocyte cultures. The results are shown as the mean ± S.E.M. No significant differences were observed between all possible comparisons (Tukey-Kramer multiple comparisons test, *P* = 0.2220).

To eliminate the possibility of unspecific binding with DL-101 mAb, we used a mix of three human B cell lines, RPMI-8226 (CD138^high^), SKW6.4 (CD138^lo^), and Ramos (CD138^neg^), which were stained with PE-conjugated B-A38, DL-101, and both mAbs (Figure 2(a)). In all three staining conditions, Ramos cells were found negative and RPMI-8226 cells were found positive. However, the binding of DL-101 to SKW6.4 was very weak suggesting that its epitope, which is on the core protein [6], might be masked by glycosaminoglycan on this cell line. We also confirmed that the DL-101 was not giving any unspecific binding in human CD19^+^ blood cells (Figure 2(b), *n* = 4) as well as for CD3 or whole CD45 populations (data not shown). Finally, to compare the binding of DL-101 and B-A38 on differentiated human B lymphocytes Alexa 647 conjugated B-A38 was combined with PE-conjugated B-B4 or PE-conjugated DL-101 (Figures 2(c) and 2(d)). The cellular profiles for five independent experiments showed that B-A38 and B-B4 mAbs were able to bind simultaneously to the CD138^+^ cells. Conversely, DL-101 and B-A38 staining have resulted in two distinct CD138^+^ cells, namely, BA^+^DL^neg^ and BA^neg^DL^+^ cells. No significant difference was observed between the frequency of BA^+^BB^+^, BA^+^DL^neg^, and BA^neg^DL^+^ cells for the five samples. However, the proportion of all BA^+^ cells decreased in the presence of DL-101 clone when comparing the same samples in Figure 1 (23.3% ± 4.3%) and Figure 2 (17.4% ± 3.0%) (Paired *t*-test, *P* = 0.0258). Overall, DL-101 mAb appeared to detect a distinct subset of human differentiated B lymphocytes.

### 3.4. Bone Marrow Plasma Cells Are DL-101 Positive

**Figure 3:**
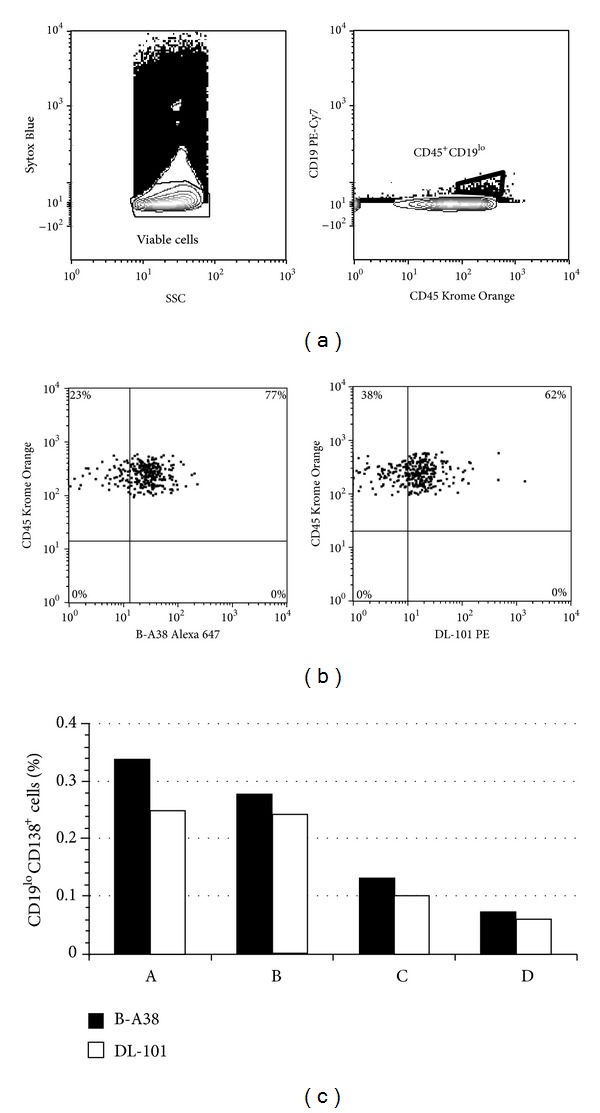
B-A38 and DL-101 are both present on bone marrow plasma cells. Four bone marrow samples were used to test the capacity of DL-101 to detect CD138^+^ cells. (a) The analysis was done on viable cells, using Sytox Blue exclusion, and then on gated CD45^+^CD19^lo^ cells, which represented less than 0.5% of total cells. (b) Profiles are showed for B-A38 and DL-101 PE-conjugated mAbs for one sample gated on CD19^lo^CD45^+^ region. The mean frequency ± S.E.M for CD138^+^ cells was 78.7% ± 1.2% for B-A38 and 59.3% ± 2.1% for DL-101. (c) Frequencies of total CD19^lo^CD138^+^ are shown for all bone marrow samples according to B-A38 and DL-101; the mean ± S.E.M. was 0.20% ± 0.06% and 0.11% ± 0.05%, respectively. No significant differences were observed between the B-A38 and DL-101 staining in these four bone marrow samples (nonparametric Kruskal-Wallis test).

To verify whether the DL-101-CD138^+^cells were present in vivo, the DL-101 staining was done on bone marrow samples (Figure 3). The viable cells were gated to select the CD45^+^ cells bearing CD19 molecules, which included the CD138^+^ plasma cells [18]. Overall, we have detected a low frequency of CD19^+^ cells (≤5%, data not shown) inside the whole cell population of bone marrow samples; the majority of the CD45^+^CD19^lo^ cells (0.5% of all cells) were as expected expressing CD138 and the frequency of these CD138^+^ cells was similar (*P* > 0.05) when using B-A38 (78.7% ± 1.2%) and DL-101 (59.3% ± 2.1%) staining indicating that plasma cells in bone marrow had both epitopes on their surface.

### 3.5. DL-101 Distinct Binding Is Specific

**Figure 4:**
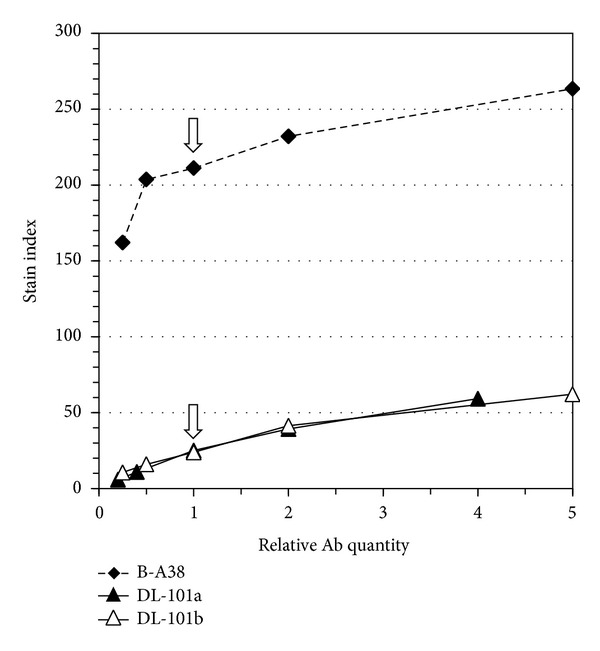
Titration of DL-101 and B-A38 staining on RPMI-8226. RPMI-8226 cells were stained with PE-conjugated DL-101 from eBioscience (DL-101a) and BioLegend (DL-101b) as well as B-A38. The manufacturer recommended volume was used as the reference (1, as indicated by arrow) and DL-101b and B-A38 were used at 0.25-, 0.5-, 1-, 2-, and 5-fold and DL-101a was used at 0.20-, 0.4-, 1-, 2-, and 4-fold. SI was calculated as described in Table 1.

We have stained RMPI-8226 cells with dilution of DL-101 and B-A38 mAbs varying from 5-fold less to up to 5-fold more quantity of antibodies as used in Table 1 and Figures 1 to 3. Titration was done for both sources of PE-conjugated DL-101 as well as B-A38 and stain index was determined as described above (Figure 4). All mAbs were staining >95% of RPMI-8226 cells. The SI profiles showed that distinctive detection of the CD138^+^ cell phenotype was not related to excessive quantity of DL-101 neither to insufficient B-A38 quantity. These results also confirm that DL-101 even in excess still have a very low SI compared to B-A38.

## 4. Discussion

In this study, we have compared the staining capacity of five anti-CD138 mAbs on human plasma cells and cells lines. We uncovered that there were significant differences between DL-101 on one side and B-A38, B-B4, and MI-15 on the other side. In fact, the anti-CD138 mAb DL-101 was weakly binding on RPMI-8226 cells, a CD138^high^ cell line, and was unable to stain SKW6.4 cells, a CD138^lo^ cell line. However, we showed that DL-101 mAb was able to stain plasma cells in bone marrow and its CD138^+^ cells detection frequency was similar to that of B-A38 mAb. Furthermore, by using DL-101 mAb to stain in vitro newly formed plasma cells, we revealed a high proportion of CD138^+^ cells, which was twofold higher than that detected with B-A38, B-B4, or MI-15 anti-CD138 antibodies. Our results also indicate that B-A38 and B-B4 can bind simultaneously to the CD138 molecules, as shown by BA^+^BB^+^ and BA^+^DL^neg^ cells frequencies (Figure 2(d)), suggesting that their targets are distinct and distant enough.

Our assays using the four anti-CD138 PE-conjugated mAbs (Figure 1) suggest that DL-101's epitope might be present on all CD138^+^ cells stained by B-B4, MI-15, and B-A38 mAbs and also on a supplemental subset (Figure 1). Besides, the use of Alexa 647 conjugated B-A38 combined with PE-conjugated DL-101 also suggests that DL-101's epitope, when present, can overlap B-A38 mAb's epitope (Figures 2(c) and 2(d)). However, the sum of BA^+^DL^neg^ and BA^neg^DL^+^ cells gave a total CD138^+^ cell frequency similar to the one observed in Figure 1 for cells stained with DL-101 alone. DL-101 and B-A38 competition or overlap is probably also responsible for the decreased frequency of B-A38^+^ cells. Furthermore, the difference in staining capacity of DL-101 compared to other anti-CD138 clones on cultured cells at day 24 as well as day 19 suggests that generated plasma cells can acquire the expression of DL-101 epitope as soon as they differentiate and that they maintain also its expression. DL-101 mAb recognizes an epitope present on the core protein and can also bind to the proteoglycan form of soluble human CD138 released by A431 cells [6]. The observed staining variation between DL-101 and B-A38 was observed on myeloma cell lines characterized for their bright or weak expression of CD138 molecules. This also suggests differences in their distribution among human B cell lines. Overall, our results suggest that the availability of DL-101's epitope could be influenced by variations in the CD138 molecules' content in heparan sulfate or chondroitin sulfate, which could result from in vitro generation of plasma cells or depend on the origin of the cell lines or even on the microenvironment and culture medium. Our observations suggest a certain level of heterogeneity inside the in vitro generated CD138^+^ plasma cells. However, whether DL-101^+^ cells represent a distinct subset specific to in vitro culture microenvironment remains to be further investigated.

The bone marrow content of CD138^+^ cells were showing similar frequency of B-A38^+^ and DL-101^+^ cells, suggesting that the majority of these cells were double positive for these epitopes. Conversely, our results showing heterogeneity were obtained with fresh samples of cell lines and in vitro generated plasma cells. Therefore, we can not exclude that the DL-101 epitope may be sensitive to freezing-thawing procedure and it remains interesting but not easy to verify the possibility of heterogeneity inside the bone marrow plasma cells.

The CD138 molecule is mainly related to plasma cells and has been rarely reported to be expressed on other leucocytes. Recently, CD138 molecules have been detected on blood monocytes from patient with active systemic lupus erythematous disease (SLE) [19]. Moreover, CD138 molecules were also present on in vitro dendritic cells generated by culturing monocytes from healthy individuals in the presence of serum from those SLE patients. In view of these results, we question whether a special subset of CD138 molecules could be associated with a strong activation of the immune system, which is an important feature of our in vitro model as well as of SLE disease [19].

## 5. Conclusion

In conclusion, DL-101 and B-A38 CD138 mAbs suggest that CD138 molecules may be different in human CD138^+^ cell lines as well as in vitro generated plasma cells. Such possible heterogeneity detected by DL-101 may be related to the in vitro culture conditions and thus B-A38, which is widely used, remains reliable. However, the single use of DL-101 to study plasma cells may cause divergences with studies using other mAbs anti-CD138 such as B-A38 to monitor plasma cells.

## Figures and Tables

**Table 1:** Comparison of anti-CD138 antibodies on RPMI-8226 cell line.

Anti-CD138 antibodies		Stain index^1^		
Clone	Fluorochrome	Unfixed^2^	Fixed cells^2^	
		<1 h	<1 h	18–20 h
1D4	FITC	3.2	2.4	0.8
B-A38	PE	85.1	78.2	14.1
	Alexa 647	118.0	78.2	21.7
B-B4	PE	32.4	16.5	3.7
DL-101	PE	1.7	3.2	1.9
MI-15	PE	24.7	17.9	5.6
	PCP CY5.5	3.4	3.7	1.3

^1^Stain index corresponds to *D*/*W* where *D* = [MFI-test − MFI-unstained] and *W* = [2 × SD-unstained] and where MFI is the median fluorescence intensity.

^2^Cells were stained and analyzed immediately without fixation (<1 h), or stained and fixed and analyzed immediately (<1 h), or kept at 4°C in the dark and analyzed after 18 to 20 hours as indicated.
